# Interactive effects of leaf pathogens and plant mycorrhizal type on plant diversity–productivity relationships

**DOI:** 10.1002/ecy.70029

**Published:** 2025-02-11

**Authors:** Nianxun Xi, Yansong Zhao, Marina Semchenko

**Affiliations:** ^1^ Hainan Baoting Tropical Rainforest Ecosystem Observation and Research Station School of Ecology, Hainan University Haikou China; ^2^ School of Life Sciences, School of Ecology, Sun Yat‐sen University Guangzhou China; ^3^ Institute of Ecology and Earth Sciences, University of Tartu Tartu Estonia; ^4^ Department of Microbial Population Biology Max Planck Institute for Evolutionary Biology Plön Germany

**Keywords:** biodiversity loss, competition, complementarity, diversity–productivity relationship, mycorrhizal fungi, plant–pathogen interactions

## Abstract

Diversity–productivity relationships can differ between forests dominated by different mycorrhizal types and be modulated by specialist and generalist pathogens. However, little is known about how these factors interact to modulate biodiversity effects. We addressed this knowledge gap with a 2‐year experiment combining the manipulation of plant richness (one, two, four, eight species) and mycorrhizal tree type (arbuscular mycorrhizal [AM] tree‐dominated; ecto‐mycorrhizal [ECM] tree‐dominated) with fungicide application for leaf pathogens (added or control). Biodiversity effects were quantified for community productivity and its two components (shoots and roots). We observed nonlinear diversity–productivity relationships, with the productivity of ECM tree‐dominated communities increasing at low to intermediate diversity and declining at the highest species richness. Foliar fungicide application reduced positive complementarity effects and increased productivity in both ECM tree monocultures as well as eight‐species mixtures. This finding suggests that the dilution effects of specialized pathogens may dominate at low diversity, while the spillover effects of generalist pathogens may become dominant at high diversity, resulting in unimodal diversity–productivity relationships. In AM tree‐dominated communities, aboveground productivity strongly increased in response to leaf pathogen suppression in eight‐species mixtures, and the release from leaf pathogens benefited most of the species that were most productive in fungicide‐treated monocultures. This agrees with the prediction that spillover effects of generalist pathogens in diverse plant communities could differentially suppress highly productive species due to the trade‐off between growth and defense. In addition, positive biodiversity effects on root production were significantly stronger in AM tree‐ than ECM tree‐dominated communities. Our results demonstrate that relationships between plant diversity and productivity can be nonlinear due to the combined effects of specialized and generalized plant–fungal interactions, depend on plant mycorrhizal type, and differ between aboveground and belowground compartments.

## INTRODUCTION

Understanding the mechanisms behind biodiversity effects on ecosystem functions is a key question in ecological research, with implications for maintaining and restoring biodiverse ecosystems (Potapov et al., 2017; Venter et al., 2016; Watson et al., 2018). In plant diversity–productivity relationships, the net biodiversity effect represents the overall impact of biodiversity on productivity, encompassing both complementarity and selection effects. It can be quantified as the difference between the observed biomass of plant communities and the expected biomass based on monoculture yields of component species (Loreau & Hector, 2001). Considerable evidence points to positive biodiversity effects arising from resource partitioning or facilitation, which are grouped as complementarity effects (Barry et al., 2019; Fargione et al., 2007). Complementarity effects decrease interspecific competition and increase resource utilization as plant diversity increases, thereby leading to greater overall plant productivity. In addition, selection effects can also contribute to positive plant diversity–productivity relationships, as the probability of including a productive species in communities increases as plant diversity increases (Loreau & Hector, 2001). Aside from these resource‐based mechanisms, plant–microbe interactions may also regulate plant diversity–productivity relationships (Mommer et al., 2018; Thakur et al., 2021). Recent studies have demonstrated that soil pathogens can be an important mediator of positive biodiversity effects (Maron et al., 2011; Schnitzer et al., 2011; van Ruijven et al., 2020; Xi et al., 2022). However, the roles of aboveground pathogens, such as leaf fungal pathogens, in driving plant diversity–productivity relationships have rarely been investigated (but see Huang et al., 2022; Seabloom et al., 2017).

**FIGURE 1:**
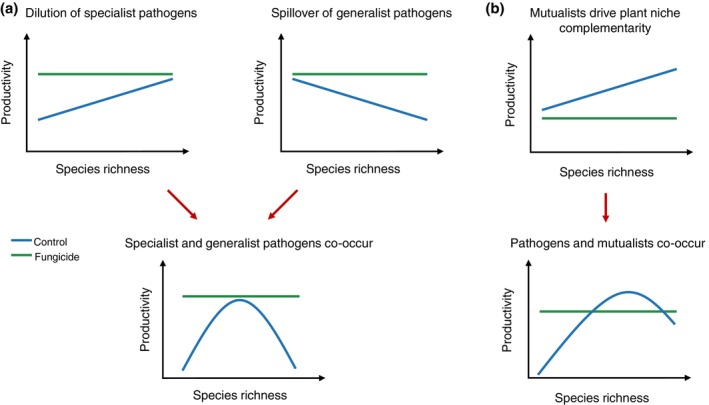
The conceptual framework of how pathogens and mutualists can modulate plant diversity–productivity relationships. (a) Impacts of pathogens. Specialist pathogens restrict productivity most in species monocultures and produce positive complementarity effect in species mixtures. Generalist pathogens suppress productivity in high‐diversity mixtures and can lead to negative complementarity effects. Simultaneous effects of specialist and generalist pathogens should lead to a unimodal relationship between plant species richness and productivity. (b) Combined impacts of mutualists and pathogens. Fungal mutualists (e.g., mycorrhizal fungi and leaf endophytes) may drive plant niche complementarity and thus improve productivity at higher diversity levels. The suppression of mutualists by fungicide application should therefore reduce productivity. Therefore, the presence of pathogens and mutualists can lead to complex and potentially nonlinear diversity–productivity relationships.

Mounting evidence suggests that when leaf pathogens are specialists, that is, infect one or few related host plants, plant diversity can reduce leaf pathogen spread and damage, because host density decreases and pathogen spread can be impeded by heterospecific neighbors (Hantsch et al., 2013, 2014; Keesing et al., 2006; Rottstock et al., 2014; Rutten et al., 2021). The dilution effects of specialist pathogens can increase host plant performance in diverse plant communities and thus may promote primary productivity (Cappelli et al., 2022; Huang et al., 2022). However, there is growing evidence that leaf pathogens with broad host ranges (i.e., generalist pathogens) are common, and that host plants vary in susceptibility to generalists (Hersh et al., 2012; Power & Mitchell, 2004; Spear & Broders, 2021). The possibility of including the more susceptible hosts increases with host diversity (selection effect on host susceptibility), and high plant diversity may favor the transmission of generalist pathogens, which are often more aggressive than specialists (e.g., necrotrophic pathogens), leading to amplification effects of pathogens and decreased community productivity (Halliday et al., 2017; Keesing et al., 2006; Power & Mitchell, 2004). We therefore predict that when plant productivity is simultaneously regulated by specialist and generalist pathogens, nonlinear diversity–productivity relationships may arise depending on the relative strength of specialist and generalist pathogen effects across diversity gradients (Figure 1a). In addition, plant productivity is not only regulated by pathogens but also by mutualists such as mycorrhizal fungi and leaf endophytes, which can improve plant nutrient acquisition and contribute to niche differentiation among species (Barry et al., 2019; Powell & Rillig, 2018; Wang et al., 2020). Therefore, fungi can both suppress and enhance productivity at different productivity levels via distinct mechanisms, leading to complex and potentially nonlinear diversity–productivity relationships (Figure 1b).

Leaf pathogen effects may vary among plant communities, with different plant mycorrhizal types. Nearly all tree species are colonized by key soil mutualists—either arbuscular mycorrhizal (AM) fungi (hosted by 94% families of angiosperms) or ecto‐mycorrhizal (ECM) fungi (hosted by only certain tree families) (Tedersoo et al., 2020; Wang & Qiu, 2006). AM trees often produce high‐quality leaves with high nitrogen and phosphorus concentrations, as well as nutrient‐rich litter, promoting plant growth rates and soil nutrient mineralization but also likely increasing susceptibility to natural enemies such as pathogens (Bennett et al., 2017; Yi et al., 2024). In contrast, ECM trees typically allocate more photosynthetic carbon to mycorrhizal fungi and defense functions, resulting in a high‐defense and low‐growth strategy (Cornelissen et al., 2001; Yi et al., 2024; Zheng et al., 2024). Therefore, leaf pathogens may contribute to positive biodiversity effects more strongly in AM than in ECM tree communities. However, the effects of plant mycorrhizal type (Dietrich et al., 2023; Ferlian et al., 2018) and leaf pathogens (Huang et al., 2022; Seabloom et al., 2017) on diversity–productivity relationships have been studied in isolation, and their interactive effects on plant diversity–productivity relationships remain unclear.

Here, we performed a 2‐year experiment to test how plant mycorrhizal type and leaf pathogens influence plant diversity–productivity relationships in tree sapling communities. We established 256 plant communities dominated by AM or ECM trees, and these communities were either sprayed with a foliar fungicide to suppress leaf pathogens or grown without fungicide as a control treatment. We addressed the key question: how do leaf pathogens influence plant diversity–biomass relationships in AM‐ versus ECM‐tree communities? Specifically, two hypotheses were tested: (1) fungi drive nonlinear plant diversity–productivity relationships, and fungicide application weakens positive biodiversity effects, and (2) foliar pathogens and mycorrhizal type interact to regulate the relationships between plant richness and productivity, so that biodiversity effects in AM tree‐dominated communities are more strongly regulated by leaf pathogens than in ECM tree‐dominated communities.

## METHODS

### Experimental design

The experiment was conducted in a shade house at Tiantong Forest Ecosystem National Observation and Research Station (121°47′ E, 29°48′ N, Ningbo, Zhejiang Province, China). The site has a subtropical monsoon climate with an average annual temperature of 16.2°C and average annual precipitation of 1374.7 mm, and the soil type is Humic Acrisols according to IUSS Working Group WRB (2022). The experiment is a full factorial design, including three factors: community type (AM or ECM tree‐dominated communities), foliar fungicide treatment (fungicide addition, control), and plant species richness (1, 2, 4 and 8 species) (Appendix S1: Figure S1). We sought to make inferences at the level of the plant community (monocultures or mixtures); thus, all treatments were applied at the scale of pots, and there were 16 replicates for each treatment combination, resulting in 256 experimental units.

Fourteen tree species common to the subtropical forests in the Tiantong region were selected for this study, including 7 AM tree species—*Daphniphyllum oldhami*, *Cinnamomum camphora*, *Neolitsea aurata*, *Hovenia acerba*, *Acer buergerianum*, *Schima superba*, and *Diospyros japonica*—and seven ECM tree species—*Aphananthe aspera*, *Cyclobalanopsis glauca*, *Cyclobalanopsis gracilis*, *Cyclobalanopsis sessilifolia*, *Lithocarpus glaber*, *Lithocarpus harlandii*, and *Quercus chenii* (Appendix S1: Table S1). These tree species cover a large range of relative abundances in the region, from the most abundant, such as *N. aurata*, *L. harlandii*, *C. sessilifolia*, and *S. superba*, to the rarest, including *C. camphora*, *L. glaber*, and *A. buergerianum*. We structured two types of plant communities, that is, AM tree‐dominated communities (the 7 AM plants and one ECM plant *C. glauca*) and ECM tree‐dominated communities (the seven ECM plants and 1 AM plant *H. acerba*) (Appendix S1: Table S1), as natural forest communities consist of both AM and ECM trees but are dominated by one mycorrhizal type.

Seeds were collected in the Tiantong forests in 2018 and germinated in April 2019. In July 2019, seedlings were transplanted into pots (27‐cm diameter, 25‐cm height) filled with field soil (average pH = 4.5, soil carbon = 44.87 g kg^−1^, soil nitrogen = 3.21 g kg^−1^, soil phosphorus = 0.26 g kg^−1^). Field soil was collected at multiple sites in the nearby natural forest where AM and ECM trees coexist, mixed thoroughly, and rocks and large organic debris were removed. Model tree communities were created with single plant species (monocultures), or with mixtures of two, four, or eight species. For both community types, there were 16 monocultures (two replicates for each plant species) and 16 combinations for each of the two‐and four‐species mixtures; species composition was determined using 16 separate random draws from the species pool (seven species and one species with the opposite mycorrhizal type). The eight‐species mixture had constant species composition, that is, seven species and one species with the opposite mycorrhizal type (16 replicates). Each community consisted of eight seedlings, which were arranged in a circle, and adjacent seedlings were spaced 7 cm apart. In the mixtures, all plant species had equal abundance. The shade house provided overhead shading but allowed free air exchange with a nearby natural forest site. Hence, natural pathogens could colonize the plants by air transfer or be recruited from soil. The pots were arranged in a grid (32 rows, 8 columns), spaced 15 cm apart, and rearranged monthly to avoid position effects.

Plant communities were left to establish mycorrhizal associations for 12 months prior to the start of fungicide application. Twelve months after plant transplanting, two fungicides, metalaxyl mancozeb and fludioxonil (Syngenta Ltd., Basel, Switzerland), were separately sprayed over the canopy of plants with an interval of 15 days for the second year, following the recommendations by the manufacturer. The control pots were kept outside during the spraying of fungicides to avoid any influence from the treatments, and they were treated with water of equal volume to the fungicide solution. Afterward, the fungicide‐treated and control pots were placed together to ensure that all plants grew under the same conditions. The two fungicides are commonly applied to suppress foliar pathogenic fungi in forests (Bagchi et al., 2014; Huang et al., 2022; Jia et al., 2020) and have been reported to have minor impacts on AM colonization in grassland species (Maron et al., 2011). At harvest, visual inspection of ECM plants confirmed that roots were colonized in both control and fungicide treatments. Moreover, the same fungicides were used in a nearby forest site where the effective suppression of leaf pathogen incidence on seedlings of eight species used in this experiment was confirmed (unpublished data).

In July 2021, tree saplings were harvested 2 years after transplanting. Shoot biomass was sorted by species in each pot. Root biomass was harvested at the pot level, because the root systems could not be clearly sorted out to individual species. Plant biomass was oven‐dried at 60°C for 48 h and weighed. Net biodiversity effects (NE) were calculated as the difference between the yield of mixtures and the average monoculture yields of component species in mixtures (Loreau & Hector, 2001). We used the partitioning approach by Loreau and Hector (2001) to calculate complementarity effects (CEs) and selection effects (SEs) on shoot biomass:

NE=CE+SE,

$$\mathrm{CE}=N\;\bar{∆{\mathrm{RY}}_{i}}\;\bar{M_{i}},$$

$$\mathrm{SE}=N\;\mathrm{cov}\left(∆{\mathrm{RY}}_{i},M_{i}\right),$$

where *i* is the component species in the mixture, *N* is the number of component species, *M*_*i*_ is the yield of species *i* in its monoculture, and ΔRY_*i*_ is the deviation from the expected relative yield of species *i* in the mixture. The observed relative yield of species *i* in the mixture is the ratio of its yield in the mixture to its yield in monoculture. The expected relative yield of species *i* is simply its seeding or planting proportion in the mixture. Complementarity effects are positive when species yield in mixtures is on average greater than expected based on their yield in monocultures. Positive selection effects arise when higher diversity in communities increases the likelihood of including species that are more productive than average, resulting in these species gaining a greater relative yield in mixtures compared with less productive species.

### Data analyses

Community biomass and biodiversity effects (NE, CE, and SE) were analyzed using mixed‐effects models in the lmerTest R package (Kuznetsova et al., 2017), with mycorrhizal type, fungicide treatment, and plant richness as fixed factors, and plant community composition as a random factor to account for the inherent variability among different plant communities. The homogeneity of variance was evaluated with the DHARMa package prior to analyses (Hartig, 2022). Plant richness was treated as a categorical variable to aid the interpretation of interactive effects, as initial model fittings indicated that the relationships between productivity and diversity were not linear. This approach allows to accurately test the effects of different factors at different levels of species richness driven by nonlinear effects of plant richness on productivity and biodiversity effects (as suggested in Klironomos et al., 2000; Schnitzer et al., 2011). If species richness were treated as a continuous variable in the full model, interactions between mycorrhizal type, fungicide treatment, and both linear and nonlinear terms (e.g., second‐order polynomial) for species richness would have to be tested, which would make interpretation of model parameters difficult. Hence, the significance of interactive effects was tested with a model including richness as a categorical variable, while nonlinear relationships in individual treatments were visualized using separate regressions between plant species richness and biomass/diversity effects for each treatment combination. We fitted linear, logarithmic, exponential, power, and second‐order polynomial functions to the relationship between plant species richness and plant biomass, as previous studies recorded these patterns (Craven et al., 2016; Klironomos et al., 2000; Schnitzer et al., 2011; Seabloom et al., 2017; Zhou et al., 2006). Those functions were also used to fit the relationships between plant species richness and net biodiversity effects. Akaike information criterion (AIC) was calculated for each model, and the model with the smallest AIC value was chosen. *t* tests were used to test whether the average values of NE, CE, and SE differed from zero. All analyses were performed in R (R Development Core Team, 2022).

## RESULTS

### Community biomass

**FIGURE 2:**
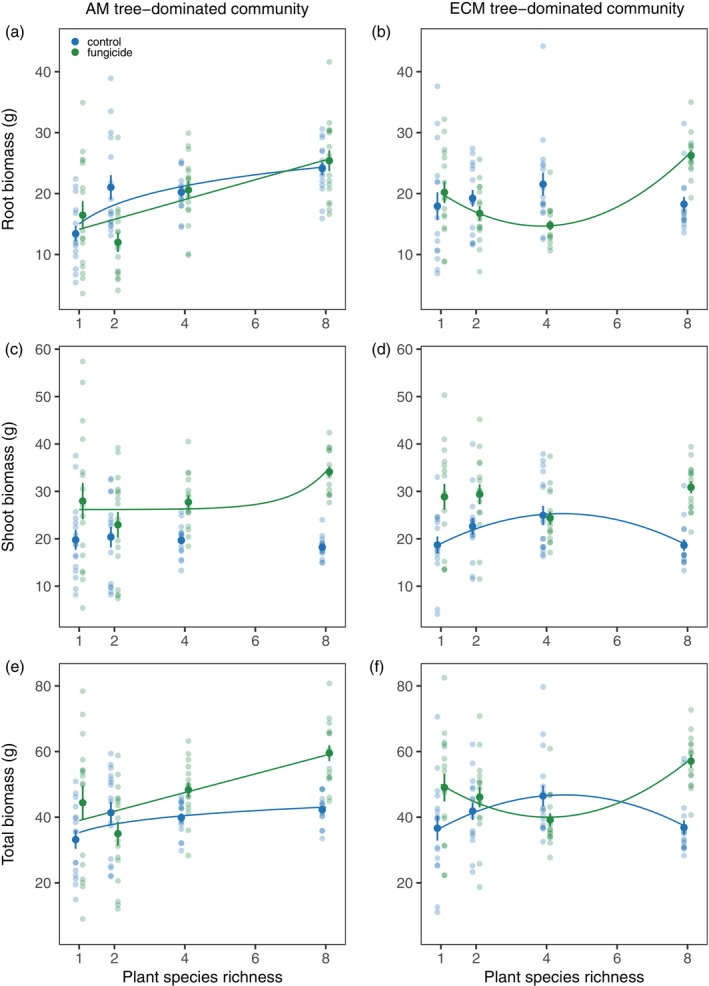
Root biomass across plant richness levels in (a) arbuscular mycorrhizal (AM) tree‐dominated and (b) ecto‐mycorrhizal (ECM) tree‐dominated communities. Shoot biomass across plant richness levels in (c) AM tree‐dominated and (d) ECM tree‐dominated communities. Total plant biomass across plant richness levels in (e) AM tree‐dominated and (f) ECM tree‐dominated communities. Solid lines indicate significant regressions at the level of *p* < 0.05. Mean values and standard errors are shown.

Overall, foliar fungicide application resulted in an increase in shoot biomass without affecting root biomass (Table 1), and this response of shoot biomass likely contributed to the observed increase in total biomass with fungicide application (Appendix S1: Figure S2). The effects of plant richness on root, shoot, and total biomass were dependent upon the fungicide treatment and mycorrhizal type (significant species richness × fungicide × mycorrhizal type interaction, Table 1). For the AM tree‐dominated communities, root biomass showed a logarithmic relationship with plant richness in the control treatment with no fungicide application (Appendix S1: Table S2)—biomass increased from monocultures to two‐species communities, but no further increase in productivity was observed with increasing species richness. In the fungicide‐added treatment, a linear positive relationship between root biomass and plant richness was observed (Figure 2a; Appendix S1: Table S2). For the ECM tree‐dominated communities, root biomass showed a unimodal relationship with plant richness in the fungicide‐added treatment, with the lowest biomass in four‐species plant communities, and there was no correlation between root biomass and species richness in the control treatment (Figure 2b; Appendix S1: Table S2).

**TABLE 1:** Results from mixed‐effects models testing effects of species richness, mycorrhizal type, and fungicide treatments and their interactions on community biomass.

Factors	df	Total biomass		Root biomass		Shoot biomass		Root:Shoot ratio	
		*F*	*p*	*F*	*p*	*F*	*p*	*F*	*p*
Species richness (SR)	3	0.64	0.591	2.17	0.103	0.03	0.992	0.90	0.449
Mycorrhizal type (M)	1	0.06	0.812	0.003	0.957	0.17	0.680	0.54	0.468
Fungicide (F)	1	**48.94**	**<0.001**	0.64	0.424	**145.51**	**<0.001**	**174.54**	**<0.001**
SR × M	3	0.41	0.744	2.05	0.116	0.23	0.874	**4.69**	**0.005**
SR × F	3	**20.30**	**<0.001**	**16.93**	**<0.001**	**13.19**	**<0.001**	**5.34**	**0.001**
M × F	1	0.04	0.845	1.00	0.319	1.47	0.227	**7.02**	**0.009**
SR × M × F	3	**7.17**	**<0.001**	**8.01**	**<0.001**	**4.85**	**0.003**	**9.05**	**<0.001**

*Note*: *F* values are given with a df. Bold types indicate significant effects at the level of *p* < 0.05.

There was no relationship between plant richness and shoot biomass of AM tree‐dominated communities in the control treatment, but shoot biomass showed a nonlinear relationship with plant richness in the fungicide treatment (Appendix S1: Table S2)—shoot biomass was the greatest in eight‐species communities and did not vary across one‐, two‐, and four‐species communities (Figure 2c). Shoot biomass of ECM tree‐dominated communities was unimodally related to species richness in the control treatment (Appendix S1: Table S2), with the greatest biomass in the four‐species communities, and in the fungicide‐added treatment, no relationship was observed (Figure 2d).

For the AM tree‐dominated communities, total biomass showed a logarithmic relationship with plant richness in the control treatment—biomass increased from monocultures to two‐species communities, and no further increase in productivity was observed with increasing species richness. In the fungicide‐added treatment, a linear positive relationship between total biomass and plant richness was observed (Figure 2e; Appendix S1: Table S2). For the ECM tree‐dominated communities, total biomass showed a unimodal relationship with plant richness in the control treatment, with the greatest biomass in four‐species plant communities, but the relationship became concave in the fungicide‐added treatment, with the lowest total biomass in the four‐species plant communities (Figure 2f; Appendix S1: Table S2).

**FIGURE 3:**
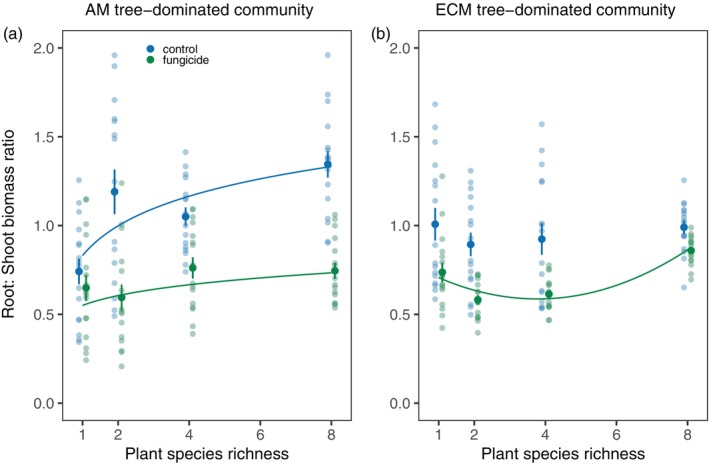
Root:Shoot biomass ratio of plant communities in (a) arbuscular mycorrhizal (AM) tree‐dominated and (b) ecto‐mycorrhizal (ECM) tree‐dominated communities. Solid lines indicate significant regressions at the level of *p* < 0.05. Mean values and standard errors are shown.

Root‐shoot ratios responded to the interaction between plant species richness, fungicide treatment, and mycorrhizal type (Table 1). Fungicide application reduced root:shoot ratios in most treatment combinations (Figure 3). For the AM tree‐dominated communities, the root:shoot ratio increased with plant species richness in the control treatment, but the fungicide application suppressed the positive relationship (Figure 3a). For the ECM tree‐dominated communities, root:shoot ratio did not change with plant species richness in the control treatment, while in the fungicide‐added treatment, it was lower in the four‐species communities compared with other species richness levels (unimodal relationship, Figure 3b).

### Biodiversity effects

**FIGURE 4:**
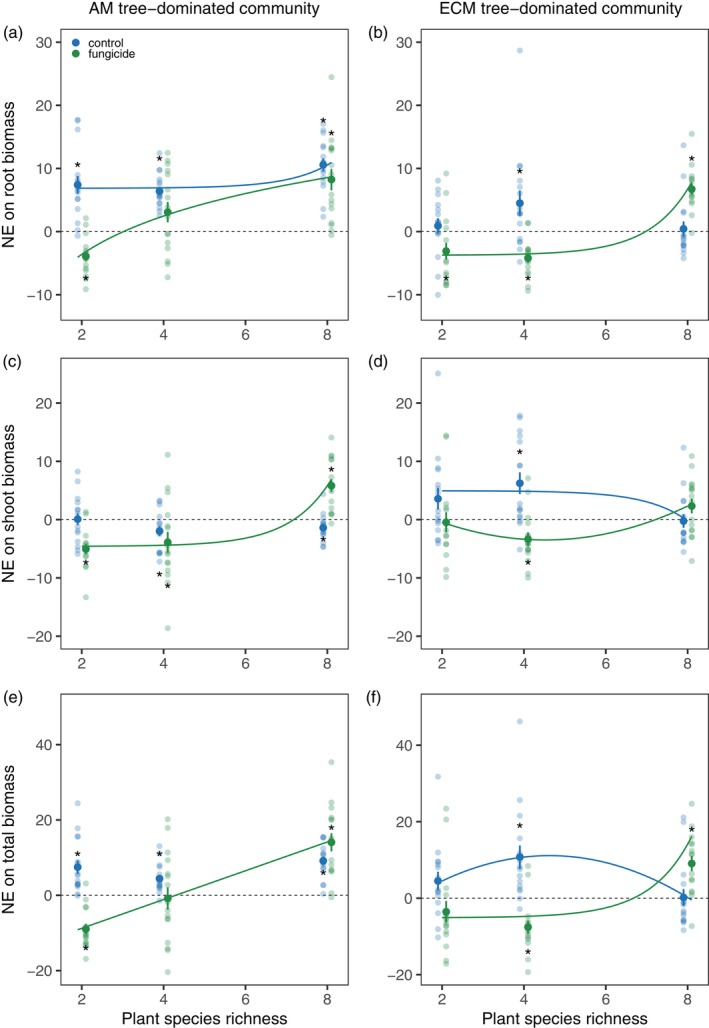
Net biodiversity effects (NE) on root biomass across plant richness levels in (a) arbuscular mycorrhizal (AM) tree‐dominated and (b) ecto‐mycorrhizal (ECM) tree‐dominated communities. NE on shoot biomass across plant richness levels in (c) AM tree‐dominated and (d) ECM tree‐dominated communities. NE on total plant biomass across plant richness levels in (e) AM tree‐dominated and (f) ECM tree‐dominated communities. Solid lines indicate significant regressions at the level of *p* < 0.05. Dashed lines indicate the value of zero. Mean values and standard errors are shown. Stars show mean values are significantly different from zero at the level of *p* < 0.05.

The effects of foliar fungicide application on net biodiversity effects (NE) depended upon plant species richness and mycorrhizal type (Table 2). For root biomass of AM tree‐dominated communities, NE was positive across plant richness levels in the control treatment (Figure 4a). Under fungicide application, NE in two‐species communities shifted from positive to significantly negative, but the effect of fungicide was reduced at higher species richness (Figure 4a). In ECM tree‐dominated communities, NE on root biomass was neutral to weakly positive in the control treatment, while fungicide application shifted NE to negative values in two‐ and four‐species communities but resulted in strongly positive NE in eight‐species communities (Figure 4b).

**TABLE 2:** Results from mixed‐effects models testing effects of species richness, mycorrhizal type, and fungicide treatments and their interactions on net biodiversity effects (NE) on total, root, and shoot biomass, and CE (complementarity effects on shoot biomass) and SE (selection effects on shoot biomass).

Factors	df	NE on total biomass		NE on root biomass		NE on shoot biomass		CE		SE	
		*F*	*p*	*F*	*p*	*F*	*p*	*F*	*p*	*F*	*p*
Species richness (SR)	2	**17.04**	**<0.001**	**25.92**	**<0.001**	0.79	0.488	0.59	0.556	1.89	0.192
Mycorrhizal type (M)	1	2.70	0.102	**38.62**	**<0.001**	3.14	0.128	**17.37**	**<0.001**	0.63	0.449
Fungicide (F)	1	**22.19**	**<0.001**	**30.03**	**<0.001**	**6.93**	**0.010**	**31.65**	**<0.001**	**26.57**	**<0.001**
SR × M	2	**4.37**	**0.014**	1.49	0.228	1.17	0.361	**3.43**	**0.034**	0.63	0.549
SR × F	2	**27.03**	**<0.001**	**17.62**	**<0.001**	**23.81**	**<0.001**	**14.66**	**<0.001**	**5.33**	**0.006**
M × F	1	0.01	0.928	**6.15**	**0.014**	**7.34**	**0.008**	0.99	0.321	**7.40**	**0.007**
SR × M × F	2	**7.26**	**0.001**	**9.87**	**<0.001**	**3.45**	**0.035**	2.79	0.064	0.94	0.394

*Note*: *F* values are given with a df. Bold types indicate significant effects at the level of *p* < 0.05.

For shoot biomass of AM tree‐dominated communities, NE was neutral to weakly negative in the control treatment, while in the fungicide‐added treatment, NE shifted from negative values at two‐ and four‐species level to strongly positive values at eight‐species level (Figure 4c). By contrast, in the ECM tree‐dominated communities, NE for shoot biomass was lower in eight‐species communities compared with two‐ or four‐species communities in the control treatment, and fungicide application caused negative NE in four‐species communities and neutral values in two‐ and eight‐species communities (unimodal relationship, Figure 4d; Appendix S1: Table S3).

As a result of combined changes in root and shoot production, NE was equally positive for the total biomass of AM tree‐dominated communities at all species richness levels in the control treatment, and the fungicide treatment shifted NE to negative values at low species richness and to more positive values in eight‐species communities (Figure 4e). In ECM tree‐dominated communities, NE was positive at four‐species communities and neutral at two‐ and eight‐species communities in the control treatment (unimodal relationship, Appendix S1: Table S3), while fungicide treatment reduced NE in four‐species communities but increased NE in eight‐species communities (Figure 4f).

**FIGURE 5:**
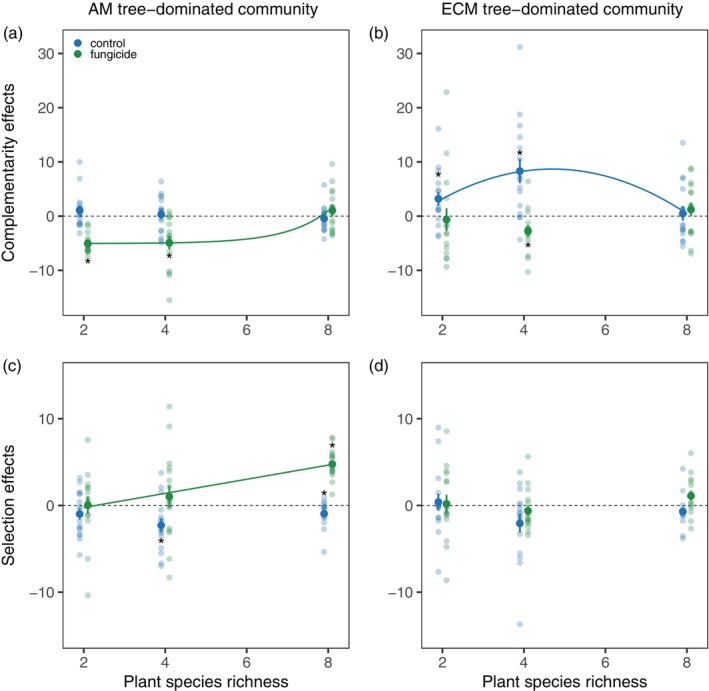
Complementarity effects (on shoot biomass) across plant richness levels in (a) arbuscular mycorrhizal (AM) tree‐dominated and (b) ecto‐mycorrhizal (ECM) tree‐dominated communities. Selection effects (on shoot biomass) across plant richness levels in (c) AM tree‐dominated and (d) ECM tree‐dominated communities. Solid lines indicate significant regressions at the level of *p* < 0.05. Dashed lines indicate the value of zero. Mean values and standard errors are shown. Stars show mean values are significantly different from zero at the level of *p* < 0.05.

Complementarity (CE) and selection effects (SE) on shoot mass varied depending upon plant richness, mycorrhizal type, and fungicide application (significant two‐way interactions in Table 2). Fungicide application shifted CE to more negative values in two‐ and four‐species communities, with no effect in eight‐species communities for both mycorrhizal types (Figure 5a,b). Fungicide application did not affect SE in ECM‐dominated communities but shifted SE from negative to positive in AM‐dominated communities (significant interaction between fungicide and mycorrhizal type treatments in Table 2; Figure 5c,d).

## DISCUSSION

Evidence is emerging that interactions with soil pathogens differ between AM and ECM trees (Bennett et al., 2017; Weigelt et al., 2021), and mycorrhizal associations influence the relationship between tree diversity and aboveground productivity (Deng et al., 2023; Ma et al., 2023). However, the role of leaf pathogens in regulating plant diversity–productivity relationships in tree communities dominated by different mycorrhizal types remains untested. Our 2‐year study manipulating leaf pathogens, tree diversity, and mycorrhizal tree composition reveals that: (1) relationships between plant diversity and productivity can be nonlinear; (2) observed responses to foliar fungicide application correspond to predictions that productivity at low and high levels of plant diversity is likely regulated by fungal specialist and generalist pathogens, respectively, and mutualist effects are most evident at intermediate levels; (3) leaf pathogens modulate biodiversity effects on productivity differently depending on the dominant mycorrhizal type, and (4) above‐ and belowground productivity show distinct responses to plant diversity and fungicide manipulations.

### Nonlinear plant diversity–productivity relationships

Our study illustrates nonlinear relationships between plant species richness and total biomass, and positive biodiversity effects on biomass were weakened under fungicide application, supporting our first hypothesis. Reduced diversity effects on aboveground tree growth upon leaf fungicide application have also been demonstrated in subtropical forests (Huang et al., 2022) and temperate grasslands (Cappelli et al., 2022). These findings contrast with another study that observed no effects of fungicide application on biodiversity effects in grasslands (Seabloom et al., 2017).

We found that fungicide effects depended on the level of tree species richness and mycorrhizal type. For AM tree communities, total biomass increased from monocultures to two‐species mixtures, and no further increase in productivity was observed with increasing species richness in the control treatment (Figure 2e). Fungicide application led to a linear relationship between plant richness and productivity, with the most pronounced increase in eight‐species mixtures (Figure 2e), reflecting the increased role of leaf pathogens in the most diverse communities. This finding is not consistent with the theory of dilution of host‐specific pathogens in species mixtures, but may reflect spillover effects of generalist pathogens (also known as amplification effect; Cappelli et al., 2020; Collins et al., 2020; Semchenko et al., 2022). More diverse plant communities can facilitate the spread of generalist pathogens (Ampt et al., 2022), which can infect more host plants, and higher biomass or denser canopy may further benefit pathogen transmission and incidence (Hersh et al., 2012).

Unlike AM trees, ECM tree communities showed unimodal plant richness–total biomass relationships in the control treatment, with the peak biomass in four‐species mixtures (Figure 2f). Fungicide application increased productivity in species monocultures and eight‐species mixtures (Figure 2f). The positive effect of fungicide in species monocultures is consistent with the prediction that plant growth is limited by high‐density specialist pathogens and that pathogen effects are diluted in species mixtures (Collins et al., 2020; Keesing et al., 2006; Mommer et al., 2018). On the other hand, increased productivity with fungicide application in eight‐species mixtures may indicate spillover effects of generalist pathogens at higher richness levels (Cappelli et al., 2020; Power & Mitchell, 2004). In the intermediate‐richness communities, fungicide application had neutral to negative effects on productivity (Figure 2f), suggesting that foliar pathogens were likely present at low densities while foliar mutualists (e.g., endophytes) may increase in abundance. The combined effects of low pathogen abundance and high mutualist abundance may drive the peak biomass observed in four‐species mixtures. Moreover, empirical evidence suggests that higher plant diversity promotes leaf pathogen diversity (Rottstock et al., 2014; Rutten et al., 2021). However, it remains poorly understood how leaf pathogen diversity interacts with plant diversity to influence productivity. Therefore, a comprehensive understanding of plant diversity–productivity relationships requires a quantitative profiling of fungal communities across different plant richness levels and test the relative roles of specialist and generalist pathogens and mutualists.

Previous studies suggest that the application of two fungicides led to a reduction in mycorrhizal colonization by the commercial AMF inoculant *Glomus irregulare* in crop plants (Jin et al., 2013), and they slightly decreased AM colonization in grassland species while the changes in root colonization did not influence plant growth (Maron et al., 2011). However, there is no definitive evidence that these fungicides suppress ECM and AM colonization in natural forests where diverse mycorrhizal communities create complex interactions with roots. Without root colonization data, we cannot completely exclude the possibility that the foliar‐applied fungicides might have disrupted plant‐mycorrhizal associations, either directly or indirectly through changes in plant physiology, potentially influencing plant nutrient uptake and diversity–productivity relationships. Future studies should directly manipulate mycorrhizal colonization to evaluate its role in influencing the effects of tree mycorrhizal types on plant diversity–productivity relationships.

### Different mechanisms underlying biodiversity effects in AM versus ECM tree communities

As AM trees are faster growing and less resistant to pathogens than ECM trees (Averill et al., 2019; Cornelissen et al., 2001), we predicted in the second hypothesis that leaf pathogens should contribute more to positive biodiversity effects in AM than ECM trees. Our results reveal a more complex pattern where biodiversity effects differ between AM and ECM trees when looking at above‐ versus belowground productivity, and fungicide application causes both negative and positive biodiversity effects depending on the level of species richness.

#### Biodiversity effects on aboveground biomass

Contrary to expectation, a positive net diversity effect on shoot mass was only found in ECM‐dominated communities at the intermediate species richness levels (Figure 4d). This effect was primarily caused by a positive complementarity effect, which was neutralized by fungicide application. This finding suggests that leaf pathogens may be the dominant factor underlying positive biodiversity effects on aboveground productivity in ECM trees. In AM‐dominated communities, neutral to slightly negative biodiversity effects on shoot mass were observed in the control treatment (Figure 4c), but fungicide application led to distinct changes in both complementarity and selection effects. In particular, complementarity effects in two‐ and four‐species mixtures became negative under fungicide addition (i.e., production in mixtures was lower than expected based on monoculture productivity) (Figure 5a). Although our study did not identify the mechanisms underlying negative complementarity effects, we speculate that fungicide application, which weakened leaf pathogen effects, may cause plant species to perform similarly (Daniel et al., 2024) and intensify competition at intermediate richness levels. This could lead to all component species performing worse than in monocultures. In particular, we found that fungicide application led to a strong increase in shoot biomass without modifying root biomass, altering the biomass ratio of roots and shoots (Figure 3), which may intensify competition for light and reduce resource partitioning. Interestingly, negative selection effects (i.e., a greater relative yield in less productive species) were replaced with strong positive selection effects (i.e., a greater relative yield in more productive species) under fungicide application at the highest species richness. This finding supports a theoretical prediction that the effects of generalist pathogens on plant performance reflect the trade‐off between growth and defense such that more productive, fast‐growing species are more susceptible to pathogen attack (Cappelli et al., 2020; Heinze et al., 2015; Parker & Gilbert, 2018). As a result, under the scenario of generalist pathogen spillover in species‐rich AM tree communities, the most productive species are most suppressed by pathogens and become more dominant under fungicide application, resulting in positive selection effects.

#### Biodiversity effects on belowground biomass

While biodiversity effects on aboveground productivity were stronger in ECM than AM trees, we found that biodiversity effects on root production were stronger in AM than ECM tree‐dominated communities (Figure 4a–d). Positive biodiversity effect was maintained under fungicide application, except for two‐species mixtures where fungicides led to negative biodiversity effects (Figure 4a). While the latter effect could be driven by intensified competition and reduced resource partitioning as discussed in the sections above, persistent more positive biodiversity effects for root biomass at higher species richness may arise from greater belowground resource complementarity among AM than ECM trees. The eight AM tree species used in this study represent seven families, while ECM trees originate from only three families, which reflects the global pattern of phylogenetic clustering of ECM symbiosis (Wang & Qiu, 2006). Therefore, AM tree communities may cover a more diverse range of resource acquisition strategies than ECM tree communities. Given observed differences between biodiversity effects on above‐ and belowground biomass compartments, we suggest that the prevalent focus on aboveground productivity alone may bias our understanding of diversity–productivity relationships, and more studies incorporating belowground biomass are needed (Eisenhauer, 2012; Grossman et al., 2018).

## CONCLUSIONS

Our study illustrates that plant diversity–productivity relationships can be nonlinear, potentially driven by combined effects of multiple factors such as specialist and generalist pathogens, mutualists, and interspecific variation representing the trade‐off between growth and defense. Dilution effects of specialist pathogens may be the key process mediating biodiversity effects at low diversity levels, while spillover effects of generalist pathogens may become more crucial at higher diversity levels. Our results also indicated that leaf pathogens regulate aboveground productivity in both AM and ECM tree communities, but the underlying mechanisms differ between mycorrhizal tree types. In particular, the productivity of ECM tree‐dominated communities matches the pattern of being co‐regulated by specialist and generalist pathogens, with the most positive biodiversity and complementarity effects observed at intermediate species richness. Because fungicides resulted in negative complementarity effects at low diversity and positive selection effects at high diversity levels in AM tree communities, we propose that leaf pathogens may maintain aboveground productivity at low to intermediate species richness levels by precluding intense aboveground competition but may limit biomass production at high species richness by differentially suppressing the dominance of highly productive species. Lastly, different patterns were observed below ground, with strong positive biodiversity effects observed in AM but not ECM tree‐dominated communities. These findings shed light on the mechanisms by which fungi and plant mycorrhizal type regulate plant diversity–productivity relationship in forests and call for future studies to consider the complexity of effects by which fungal pathogens can modulate plant growth and resource competition at different levels of plant diversity. Our study has strong implications for managing systems such as tree plantations where fungicides are often used to remove pathogens—high plant diversity is necessary to benefit ecosystem functions in these systems.

## AUTHOR CONTRIBUTIONS

Nianxun Xi designed the study. Nianxun Xi and Yansong Zhao conducted the experiment and analyzed the data. Nianxun Xi and Marina Semchenko worked on the conceptual framework and wrote the manuscript. All authors contribute to data interpretations.

## CONFLICT OF INTEREST STATEMENT

The authors declare no conflicts of interest.

## Supporting information


Appendix S1.

## Data Availability

Data (Xi et al., 2025) are available in Dryad at https://doi.org/10.5061/dryad.fbg79cp3b.
