# Supplementary material for: Interactive effects of leaf pathogens and plant mycorrhizal type on plant diversity–productivity relationships
Source: Ecology. 2025 Feb 11;106(2):e70029. doi: 10.1002/ecy.70029 (PMC11814911; doi:10.1002/ecy.70029)
Supplement: Supplementary file 1 — Appendix S1. [file ECY-106-e70029-s001.pdf]

Appendix S1

Journal: Ecology

Title: Interactive effects of leaf pathogens and plant mycorrhizal type on plant diversity–productivity relationships

Authors: Nianxun Xi, Yansong Zhao, Marina Semchenko

Table S1 Tree species used in the present study. Information includes tree mycorrhizal type, life form and whether each species was used in AM- or ECM-dominated or both communities.

Tree species	Mycorrhizal type	Life form	Community group
Daphniphyllum oldhami	AM	Evergreen	AM
Cinnamomum camphora	AM	Evergreen	AM
Neolitsea aurata	AM	Evergreen	AM
Acer buergerianum	AM	Deciduous	AM
Schima superba	AM	Evergreen	AM
Diospyros japonica	AM	Deciduous	AM
Hovenia acerba	AM	Deciduous	Both
Cyclobalanopsis glauca	ECM	Evergreen	Both
Aphananthe aspera	ECM	Deciduous	ECM
Quercus chenii	ECM	Evergreen	ECM
Cyclobalanopsis gracilis	ECM	Evergreen	ECM
Cyclobalanopsis sessilifolia	ECM	Evergreen	ECM
Lithocarpus glaber	ECM	Evergreen	ECM
Lithocarpus harlandii	ECM	Evergreen	ECM

Table S2 Results of fits of logarithmic, exponential, power, linear and 2-order polynomial for relationships of species richness and total/ root/ shoot biomass under different treatments. SR = Species richness.

	R^2	P	AIC
**(1) Total biomass ~ SR**			
AM, Control			
Logarithmic	0.0740	0.01687	472.91
Exponential	0.0196	0.13790	476.56
Power	0.1101	0.00429	473.16
Linear	0.0477	0.04578	474.70
2-order polynomial	0.0546	0.06743	475.19
AM, Fungicide			
Logarithmic	0.1451	0.00111	535.16
Exponential	0.1886	0.00020	531.82
Power	0.1493	0.00095	533.79
Linear	0.2015	0.00012	530.80
2-order polynomial	0.1981	0.00044	532.03
ECM, Control			
Logarithmic	-0.0138	0.70880	509.36
Exponential	0.0112	0.19500	507.76
Power	-0.0021	0.35470	509.39
Linear	-0.0142	0.73390	509.39
2-order polynomial	0.0780	0.03137	504.25
ECM, Fungicide			
Logarithmic	0.0051	0.25430	516.84
Exponential	0.1447	0.00113	507.16
Power	0.0158	0.16100	516.68
Linear	0.0520	0.03882	513.75
2-order polynomial	0.2039	0.00036	503.54
**(2) Root biomass ~ SR**			
AM, Control			
Logarithmic	0.2740	0.00001	406.52
Exponential	0.1409	0.00131	417.29
Power	0.3286	<0.00001	407.77
Linear	0.2201	0.00005	411.10
2-order polynomial	0.2405	0.00008	410.36
AM, Fungicide			
Logarithmic	0.2030	0.00011	446.27
Exponential	0.2026	0.00011	446.30
Power	0.2122	0.00008	444.34
Linear	0.2504	0.00002	442.34
2-order polynomial	0.2386	0.00009	444.30

ECM, Control			
Logarithmic	-0.0133	0.68070	433.49
Exponential	-0.0095	0.52500	433.25
Power	0.0031	0.27810	433.51
Linear	-0.0161	0.99400	433.67
2-order polynomial	0.0102	0.27390	432.95
ECM, Fungicide			
Logarithmic	0.0645	0.02417	420.32
Exponential	0.3540	<0.00001	396.62
Power	0.0607	0.02786	419.41
Linear	0.1819	0.00026	411.73
2-order polynomial	0.4426	<0.00001	388.13
**(3) Shoot biomass ~ SR**			
AM, Control			
Logarithmic	-0.0067	0.44870	420.71
Exponential	-0.0014	0.34330	420.38
Power	-0.0160	0.91640	420.71
Linear	-0.0026	0.36440	420.45
2-order polynomial	-0.0172	0.62910	422.34
AM, Fungicide			
Logarithmic	0.0465	0.04791	484.49
Exponential	0.0931	0.00818	481.28
Power	0.0908	0.00893	483.92
Linear	0.0819	0.01251	482.07
2-order polynomial	0.0856	0.02443	482.77
ECM, Control			
Logarithmic	-0.0151	0.79990	438.72
Exponential	0.0276	0.10000	435.97
Power	-0.0068	0.45060	438.74
Linear	-0.0105	0.55930	438.43
2-order polynomial	0.1130	0.00964	431.05
ECM, Fungicide			
Logarithmic	-0.0160	0.91700	452.79
Exponential	0.0151	0.16620	450.81
Power	-0.0086	0.50020	452.79
Linear	-0.0105	0.55790	452.45
2-order polynomial	0.0399	0.10810	450.14

Table S3 Results of fits of linear, 2-order polynomial, logarithmic, exponential and power models for relationships of species richness and net biodiversity effects (NE) total/ root/ shoot biomass under different treatments. SR = Species richness.

		R^2	P	AIC
**(1) NE on total biomass ~ SR**				
AM, Control				
	Logarithmic	-0.0059	0.39970	305.99
	Exponential	0.0526	0.06373	303.12
	Power	0.0037	0.28430	305.95
	Linear	0.0131	0.20920	305.08
	2-order polynomial	0.0857	0.05010	302.36
AM, Fungicide				
	Logarithmic	0.5014	<0.00001	355.02
	Exponential	0.4560	<0.00001	359.21
	Power	0.3130	0.00002	353.56
	Linear	0.5162	<0.00001	353.58
	2-order polynomial	0.5057	<0.00001	355.55
ECM, Control				
	Logarithmic	0.0051	0.27070	369.99
	Exponential	0.0826	0.02681	366.09
	Power	0.0182	0.17810	370.14
	Linear	0.0315	0.11870	368.70
	2-order polynomial	0.1221	0.02009	364.93
ECM, Fungicide				
	Logarithmic	0.1961	0.00095	362.22
	Exponential	0.3656	<0.00001	350.86
	Power	0.1609	0.00276	359.77
	Linear	0.2716	0.00009	357.49
	2-order polynomial	0.3766	0.00001	350.96
**(2) NE on root biomass ~ SR**				
AM, Control				
	Logarithmic	0.0611	0.04981	283.33
	Exponential	0.1254	0.00780	279.92
	Power	0.0722	0.03615	283.19
	Linear	0.0898	0.02184	281.84
	2-order polynomial	0.1155	0.02375	281.41
AM, Fungicide				
	Logarithmic	0.4334	<0.00001	306.54
	Exponential	0.2923	0.00004	317.22
	Power	0.4609	<0.00001	307.26
	Linear	0.4027	<0.00001	309.08
	2-order polynomial	0.4243	<0.00001	308.25

ECM, Control				
	Logarithmic	-0.0206	0.82490	315.75
	Exponential	0.0090	0.23870	314.34
	Power	-0.0211	0.86810	315.75
	Linear	-0.0138	0.55090	315.43
	2-order polynomial	0.0486	0.12260	313.33
ECM, Fungicide				
	Logarithmic	0.3980	<0.00001	292.91
	Exponential	0.6001	<0.00001	273.27
	Power	0.3863	<0.00001	289.73
	Linear	0.5004	<0.00001	283.96
	2-order polynomial	0.5982	<0.00001	274.44
**(3) NE on shoot biomass ~ SR**				
AM, Control				
	Logarithmic	0.0119	0.21720	255.69
	Exponential	-0.0170	0.64710	257.07
	Power	0.0016	0.30480	255.66
	Linear	-0.0008	0.33200	256.30
	2-order polynomial	0.0259	0.20830	255.95
AM, Fungicide				
	Logarithmic	0.3823	<0.00001	305.62
	Exponential	0.4632	<0.00001	298.89
	Power	0.2172	0.00050	303.55
	Linear	0.4393	<0.00001	300.98
	2-order polynomial	0.4545	<0.00001	300.60
ECM, Control				
	Logarithmic	0.0301	0.12370	325.67
	Exponential	0.1022	0.01527	321.96
	Power	0.0360	0.10380	325.82
	Linear	0.0583	0.05396	324.25
	2-order polynomial	0.1095	0.02769	322.51
ECM, Fungicide				
	Logarithmic	0.0173	0.18320	309.39
	Exponential	0.0969	0.01781	305.34
	Power	0.0274	0.13410	309.24
	Linear	0.0462	0.07692	307.96
	2-order polynomial	0.1224	0.01991	304.90

Table S4 Results of fits of linear, 2-order polynomial, logarithmic, exponential and power models for relationships of species richness and CE/ SE (shoot biomass) under different treatments. SR = Species richness.

	R^2	P	AIC
**(1) Complementarity effects ~ SR**			
AM, Control			
Logarithmic	0.0210	0.16320	246.17
Exponential	0.0111	0.22240	246.65
Power	0.0191	0.17290	246.17
Linear	0.0196	0.17030	246.24
2-order polynomial	-0.0008	0.38230	248.17
AM, Fungicide			
Logarithmic	0.2858	0.00005	270.49
Exponential	0.3781	0.00000	263.85
Power	0.1894	0.00117	269.48
Linear	0.3407	0.00001	266.66
2-order polynomial	0.3643	0.00001	265.85
ECM, Control			
Logarithmic	0.0026	0.29530	331.05
Exponential	0.0969	0.01777	326.28
Power	0.0168	0.18610	331.16
Linear	0.0329	0.11370	329.57
2-order polynomial	0.1669	0.00618	323.35
ECM, Fungicide			
Logarithmic	-0.0057	0.39590	314.45
Exponential	0.0301	0.12370	312.71
Power	0.0132	0.20830	314.39
Linear	0.0071	0.25380	313.83
2-order polynomial	0.0308	0.18580	313.62
**(2) Selection effects ~ SR**			
AM, Control			
Logarithmic	-0.0217	0.96610	228.54
Exponential	-0.0048	0.38310	227.74
Power	-0.0212	0.87940	228.54
Linear	-0.0188	0.71580	228.40
2-order polynomial	0.0222	0.22660	227.37
AM, Fungicide			
Logarithmic	0.1871	0.00126	271.07
Exponential	0.2033	0.00077	270.10
Power	0.1662	0.00236	270.81
Linear	0.2067	0.00069	269.89
2-order polynomial	0.1935	0.00298	271.63

ECM, Control			
Logarithmic	-0.0052	0.38870	262.04
Exponential	-0.0215	0.92400	262.82
Power	-0.0149	0.58130	262.02
Linear	-0.0158	0.60550	262.54
2-order polynomial	0.0413	0.14570	260.72
ECM, Fungicide			
Logarithmic	-0.0058	0.39700	247.34
Exponential	0.0211	0.16290	246.05
Power	0.0047	0.27430	247.32
Linear	0.0044	0.27800	246.86
2-order polynomial	0.0119	0.28720	247.44

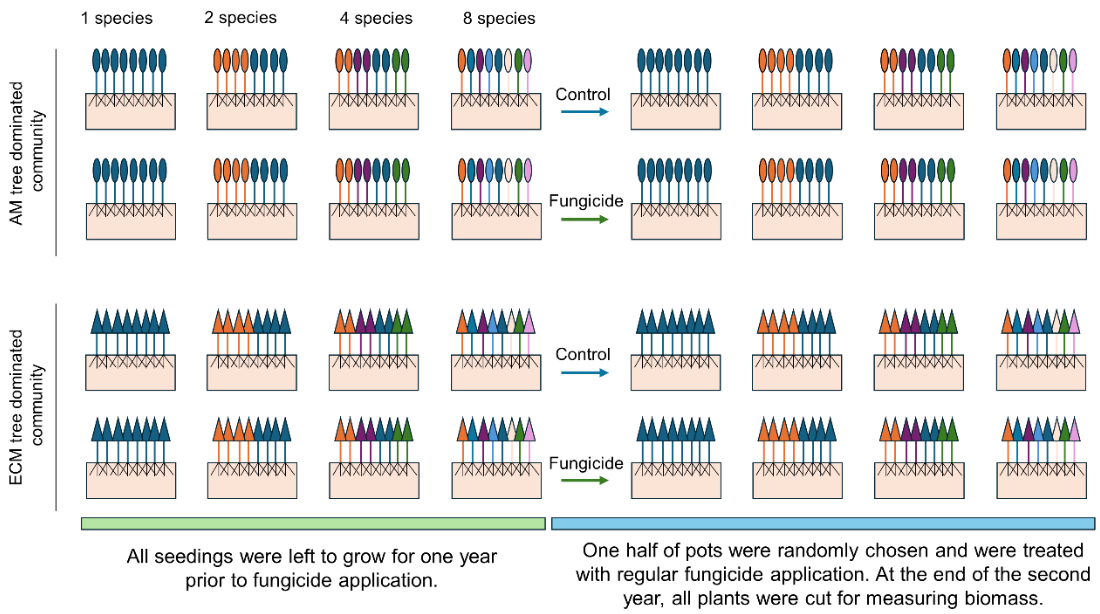

Figure S1 An illustration of experimental design. The experiment is a full factorial design, including three factors: community type (AM- or ECM-tree dominated communities), fungicide treatment (fungicide addition, control) and plant species richness (1, 2, 4 and 8 species). Plant communities were left to establish mycorrhizal associations for 12 months prior to the start of fungicide application. The two fungicides, metalaxyl mancozeb and fludioxonil, were separately sprayed over the canopy of plants with an interval of 15 days for the second year.

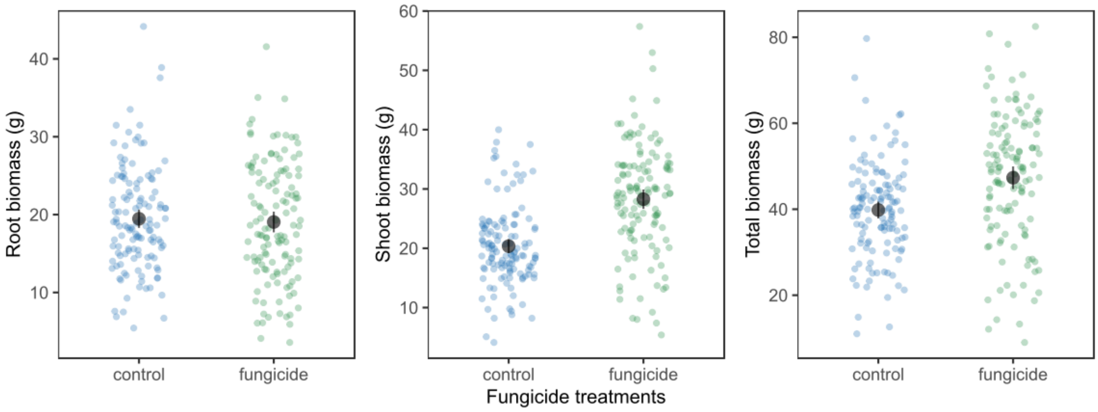

Figure S2 (a) Root biomass, (b) shoot biomass and (c) total biomass under control and fungicide treatments. The black dots and bars indicate the average values and 95% confidence intervals.
